# Retraction: Sensory Coding by Cerebellar Mossy Fibres through Inhibition-Driven Phase Resetting and Synchronisation

**DOI:** 10.1371/annotation/0b18c274-2427-482d-add4-aa20001c0e98

**Published:** 2012-04-16

**Authors:** Tahl Holtzman, Henrik Jöntell

The corresponding author requests the retraction of this article as the work was published without two key contributors. These contributors disagree with the publication of the article as it uses data that they had generated in part with the corresponding author but were not yet ready to publish themselves. The corresponding author sincerely apologises to the editor and readership for this misjudgement.

